# Changes in Hormonal Profile and Body Mass Index in Women with Polycystic Ovary Syndrome After Probiotic Intake: A 12-Week Placebo-Controlled and Randomized Clinical Study

**DOI:** 10.3390/nu17030405

**Published:** 2025-01-23

**Authors:** Iwona Szydłowska, Jolanta Nawrocka-Rutkowska, Amalia Gorzko, Hubert Pawłowski, Andrzej Starczewski, Małgorzata Szczuko

**Affiliations:** 1 Department of Gynecology, Endocrinology and Gynecological Oncology, Pomeranian Medical University in Szczecin, 71-252 Szczecin, Poland; iwona.szydlowska@pum.edu.pl (I.S.); jolanta.nawrocka.rutkowska@pum.edu.pl (J.N.-R.); andrzejstarcz@tlen.pl (A.S.); 2 University Clinical Hospital No. 1, 71-252 Szczecin, Poland; 3 Klinik für Anästhesiologie und Intensivmedizin, Heisenberg-Professur für Medizinische Risikokompetenz & Evidenzbasiertes Entscheiden, Charité—Universitätsmedizin Berlin, 10117 Berlin, Germany; hubert.pawlowski@charite.de; 4 Department of Human Nutrition and Metabolomics, Pomeranian Medical University in Szczecin, 71-460 Szczecin, Poland; malgorzata.szczuko@pum.edu.pl; 5 Department of Human Nutrition and Bromatology, Pomeranian Medical University, 71-210 Szczecin, Poland

**Keywords:** probiotics, PCOS, hormones, SHBG, BMI

## Abstract

Introduction: The beneficial effect of probiotics on the improvement of carbohydrate and lipid metabolism, as well as body mass index (BMI), has been demonstrated in various patient groups. We aimed to investigate the effect of a multi-strain probiotic on the hormonal balance of women with PCOS. Ethical approval was obtained from the Bioethical Committee. Methods: The study was designed as a 12-week, randomized, double-blind, placebo-controlled clinical study. The probiotic SanProbi^®^ Barrier capsules, which contain a unique composition of nine probiotic bacteria strains (*Lactobacillus and Biffidobacterium*), were used in the study. The mean age of the study participants was a mean of 28.42 ± 5.62 years. A total of 50 women with PCOS, diagnosed based on Rotterdam ESHRE criteria, were included in the study. Among them, 25 women were randomized to a placebo group, and 25 to a probiotic group. Results: A comparison of changes in individual hormone levels between groups confirmed statistically significant differences for TSH, androstenedione, SHBG, and BMI. In the case of LH, the statistical significance of the difference in delta change in the probiotic group was demonstrated with the use of a one-tailed test. Conclusions: Probiotic supplementation may serve as an alternative supporting treatment, especially in the phenotype of women with a high FAI index. Probiotic therapy is also effective in reducing BMI in overweight women with PCOS

## 1. Introduction

Polycystic ovary syndrome (PCOS) is the most common endocrinopathy in female patients of reproductive age. It is estimated that it concerns about 10–15% of the population [1,2,3]. This condition not only causes bothersome symptoms but also poses long-term health risks. Excessive androgen levels, ovarian ultrasound image, and anovulation are included in the criteria of disease [3]. The long-term health burden of PCOS includes hyperinsulinemia and insulin resistance (IR), as well as overweight symptoms and obesity [4,5]. Fat cells (adipocytes) produce peptide hormones such as resistin and leptin, as well as proinflammatory cytokines (IL-beta, TNF-alpha), contributing to the occurrence of chronic inflammation [6]. IR plays a fundamental role in metabolic dysfunction, including hypertension, dysglycemia, and dyslipidemia, which, in turn, lead to serious health consequences. These pathologies are linked with metabolic syndrome, type 2 diabetes and increased risk of cardiovascular diseases and chronic kidney disease (CKD). Dysfunction of the hypothalamus–pituitary–ovarian axis is characteristic of the disease [7]. It is estimated that the androgenic environment of the ovary stimulates luteinizing hormone (LH) production in the anterior pituitary and, thus, promotes later production of androgens by thecal cells in ovaries. Adipose tissue conversion changes these androgens into estrone, a hormone that intensifies LH production. Insulin acts synergistically with the luteinizing hormone to increase androgen production (hyperinsulinemia thus exacerbates hyperandrogenism). It reduces hepatic synthesis of the testosterone-binding protein (SHBG), which causes testosterone to circulate in a free, active form [8]. Recent research revealed that the Anti-Müllerian hormone (AMH) levels also are positively correlated with elevated LH levels and the LH/FSH (follicle stimulating hormone) ratio in patients with PCOS [9]. Disturbed LH/FSH ratio is one mechanism resulting in progressive metabolic disorders and fertility [9,10].

The gut microbiota was assessed as one of the pivotal elements in endocrine regulation of the body. Dysbiosis may constitute a background for many metabolic and cardiovascular abnormalities [11,12]. It also leads to a decline in estrogen-metabolizing bacteria and consequently decreases concentration of circulating estrogens, which may have serious health consequences [13]. Current research allows us to take innovative treatments into consideration, in which the homeostasis of the body could be achieved through the use of “good” gut microbiota. Modified dietary interventions, including the introduction of selected bacterial cultures that promote the maintenance of a healthy physiological flora in the gastrointestinal tract, could contribute to the regulation of hormonal balance [12,14,15]. This is particularly important for patients with polycystic ovary syndrome, which is often associated with the aforementioned disorders. Probiotics and synbiotics have gained attention as potential treatments for PCOS by modulating the gut microbiota. However, the authors of the 2024 review stated that more research is needed to determine the exact mechanisms, optimal bacterial strains, doses, and duration of this treatment [16]. Thus, much of the research in recent years underlie the role of probiotics intake as having favorable influence on gut microbiota, and thereby offering benefits in the prevention and treatment of several diseases [17,18]. Bacterial strains introduced in multispecies probiotic SanProbi^®^ Barrier, has been documented as effective in reducing body mass index (BMI), glucose homeostasis and lipid profiles in females with PCOS [19,20,21]. Sanprobi Barrier is one of the products that supports and maintains the functioning of the intestinal barrier, inhibits proinflammatory cytokines, and reduces the endotoxin load [22,23,24].

To date, the literature has primarily focused on studies analyzing changes in individual hormone levels following probiotic administration. The results vary in statistical significance and involve different groups of female patients, including both healthy individuals and those with various comorbidities [11,25,26]. The often-conflicting results of studies highlight the need for further research on this subject. To address this gap in the context of the Polish population, our study focuses on a specific analysis of the effects of probiotics on various hormone concentration levels and BMI in a homogeneous group of Polish patients with PCOS. This approach concentrates on a significant group of patients for whom targeted treatments may be developed in the future. We assessed whether the regulation of gut microbiota using probiotics, containing live bacterial strains, leads to an improvement in hormonal parameters and body mass index (BMI) in patients with PCOS. We compared hormone concentrations and BMI before and after a 12-week intervention with probiotics.

## 2. Materials and Methods

The study was designed as a 12-week, randomized, double-blind, placebo-controlled clinical study. Ethical approval was obtained from the Bioethical Committee. Clinical Trial number for the study: NCT 06761625. The study group consisted of 50 women with PCOS. The patients with comorbidities, such as diabetes mellitus type I and II, heart diseases, liver diseases, hypertension, and hypothyroidism were excluded from the study. 

### 2.1. Study Group

**Figure 1:**
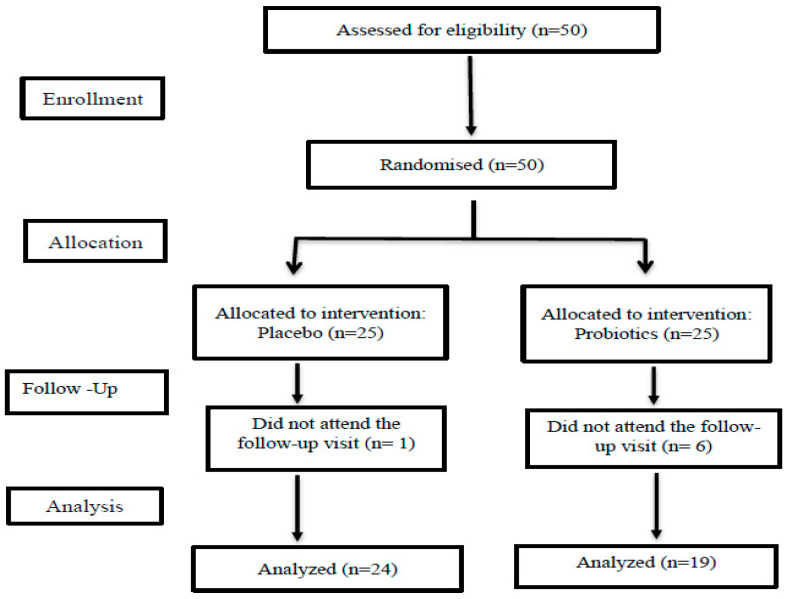
The flowchart of the recruitment, randomization, and allocation in the study.

A total of 50 women with PCOS, diagnosed based on Rotterdam ESHRE criteria, were initially invited to participate. Ultrasound pictures of ovaries were assessed with an Alpinion X-CUBE 70 imaging system; 11 Mhz endovaginal transducer. Patients discontinued hormonal medications for at least 3 months prior to treatment, and any other medications that could affect the study outcomes for at least 1 month. Patients with systemic conditions associated with PCOS that could alter the results were excluded from the study. The weight and height were measured before and after the 12-week intervention. BMI was calculated according to the standard calculation: BMI = body weight (kg)/height (m^2^) [27]. Study participants were between 19 and 42 years old (mean 28.42 ± 5.62 years). Among them, 25 women were randomized to a placebo group, and 25 to a probiotic group. Finally, the study finished with 19 women from the probiotic group and 24 women from the placebo group. Six participants from the probiotic group and one from the placebo group did not attend the follow-up visits after 12 weeks. Figure 1 illustrates the flowchart of recruitment, randomization, and allocation in the study.

### 2.2. Probiotic Intervention

Participants were randomly assigned to receive probiotics or a placebo. The probiotic group received SanProbi^®^ Barrier capsules, each containing 5 × 10^8^ CFU, totaling 1.0 × 10^9^ CFU per day in two doses. This preparation included nine bacterial strains: Bifidobacterium bifidum W23, Bifidobacterium lactis W52, Lactobacillus acidophilus W37, Lactobacillus brevis W63, Lactobacillus casei W56, Lactobacillus salivarius W24, Lactococcus lactis W19, Bifidobacterium lactis W51, and Lactococcus lactis W58.

The placebo group received identical-looking capsules filled mainly with maize starch and maltodextrins. All participants were instructed to take one capsule two times daily for 12 weeks, maintaining a typical diet and routine physical activity while reporting any side effects.

### 2.3. Biochemical Tests

The serum samples were obtained on days 3–5 (early follicular phase) of the menstrual cycle. In the laboratory studies, the following hormones were measured before and after 12-week intervention: Testosterone, Androstenedione, 17-hydroxyprogesterone (17(OH)P), follicle stimulating hormone (FSH), luteinizing hormone (LH), estradiol (E_2_), prolactin (PRL), thyroid stimulating hormone (TSH), thyroxine (T4), and sex hormone binding globulin (SHBG). These hormones were measured in venous blood samples using the ELISA method.

### 2.4. Statistical Analysis

The adopted level of significance was *p* < 0.05. The normality of the measurement distributions was assessed using the Shapiro–Wilk test, while the homogeneity of variances was evaluated with Levene’s test. Since, in most cases, the distributions of results were not normal and/or did not exhibit homogeneity of variances, the non-parametric Wilcoxon test for dependent groups was used to compare baseline and final measurements and the Mann–Whitney U test was used for comparisons between the probiotic and placebo groups. The t-test for independent groups was used to compare anthropometric characteristics between the experimental and control groups. The statistical analysis was performed using the Statistica 13.0 (StatSoft, Krakow, Poland) software package.

## 3. Results

The baseline characteristics of the groups revealed no differences in basis anthropometric data between placebo and probiotic groups (Table 1). After 12-week intervention, statistically significant differences in biochemical parameters were observed between groups (Table 2).

A comparison of the mean concentrations of the studied hormones after 12 weeks, both within the study group and the control group (final measurements) with the average baseline measurement results reveals that statistically significant changes occurred only in the probiotic group concerning the following hormones and BMI (Table 2):

- LH (decrease in concentration; *p* = 0.0218);
- TSH (decrease in concentration; *p* = 0.0044);
- Androstenedione (decrease in concentration; *p* = 0.0038);
- SHBG (increase in concentration; *p* = 0.0012);
- BMI (decrease; *p* = 0.0198).

**Figure 2:**
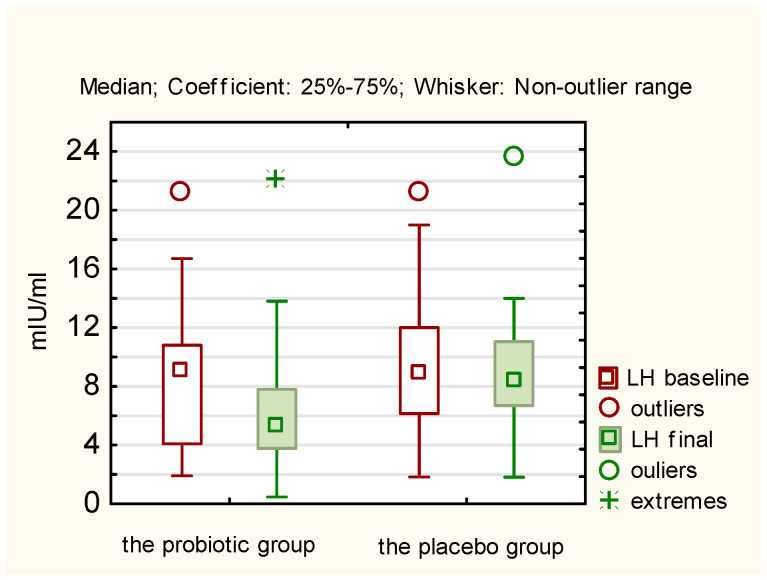
Initial and final LH concentration (mIU/mL) in the probiotic and placebo groups.

**Figure 3:**
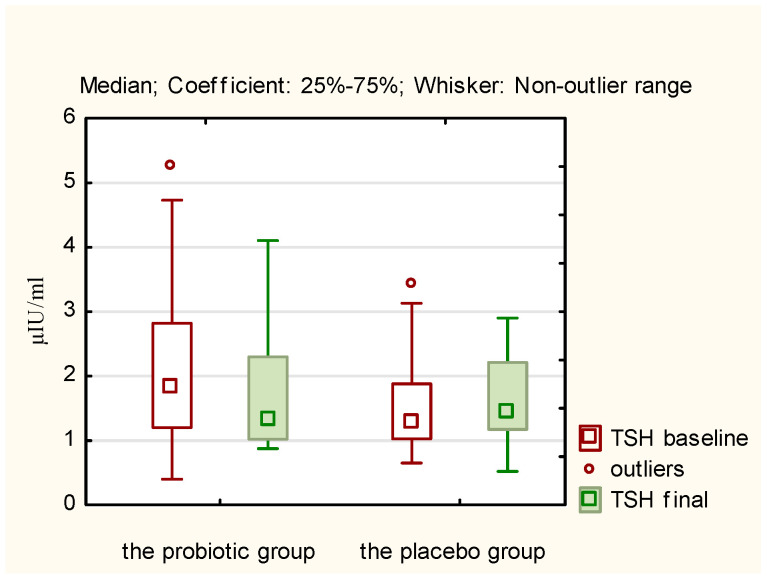
Initial and final TSH concentration (μIU/mL) in the probiotic and placebo groups.

**Figure 4:**
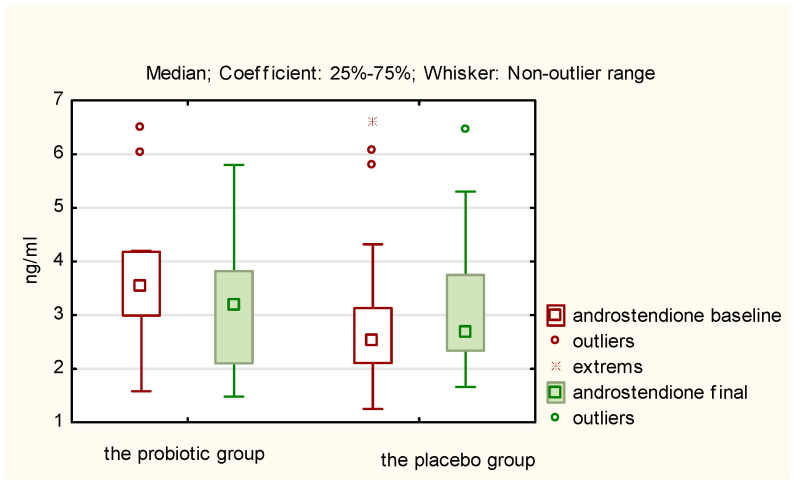
Initial and final androstenedione concentration (ng/mL) in the probiotic and placebo groups.

**Figure 5:**
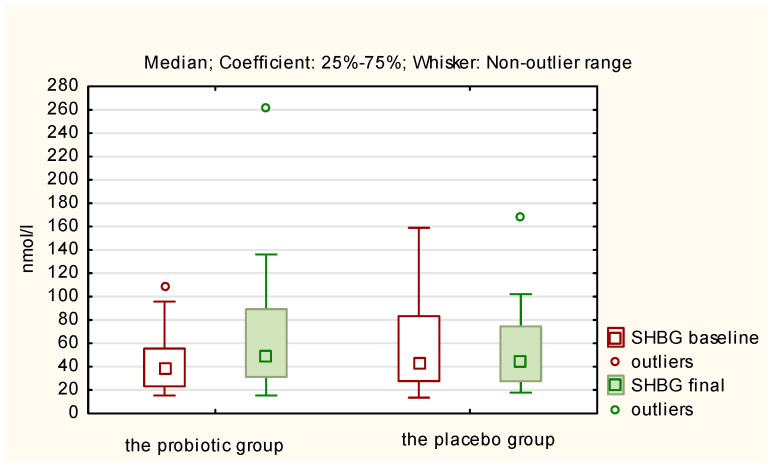
Initial and final SHBG concentration (nmol/l) in the probiotic and placebo groups.

The above results of hormones in both the probiotic and placebo groups are presented in Figure 2, Figure 3, Figure 4 and Figure 5. They illustrate statistically significant and visible changes after probiotic therapy in LH, TSH, androstendione, and SHBG concentrations. We observed that LH, TSH, and androstenedione levels decreased, while SHBG levels increased in the probiotic group.

A comparison of the delta changes (differences between baseline and final measurements) for individual hormones in the study group, with the delta changes observed in the placebo group, confirmed that there are statistically significant differences between the study group and the control group for TSH, androstenedione, and SHBG (Table 3). In the case of LH, the statistical significance of the difference between the delta change in the probiotic group and the delta change in the control group can be demonstrated with the use of a one-tailed test.

## 4. Discussion

The relationship between the gut microbiome and endocrine system disorders has been extensively studied [19,21,28]. By manipulating the microbial composition and introducing probiotic supplementation, it is possible to modulate plasma concentrations of various substances, including hormones, thereby supporting the conventional treatment of endocrine diseases [22,25]. The current review of meta-analyses indicated that, although there are various mechanisms by which probiotics may benefit women with PCOS, the magnitude of the effect was not clinically significant [21]. Both synbiotics and probiotics administration were associated with significant improvements in body mass index (BMI), revealed Cozzolino et al [29]. Supplementation with synbiotics had a beneficial effect on hirsutism scores and supplementation with synbiotics and probiotics significantly increased SHBG synthesis. Nevertheless, these findings were based on low- to very low-quality evidence according to the Grading of Recommendations Assessment, Development and Evaluation assessment tool [29].

Regarding hormonal changes, only levels of dehydroepiandrosterone sulfate were significantly reduced following probiotic supplementation; however, the certainty of the evidence was rated as low [21]. Kaur et al. observed a significant decrease in BMI, an improvement in the LH/FSH ratio, and a reduction in total testosterone levels after 6 months of multi-strain probiotic supplement in PCOS women [26]. In our study, we did not observe any changes in dehydroepiandrosterone sulfate (DHEA-S) nor testosterone levels. A significant drop in androstenedione and LH concentrations were noted by our team. Additionally, an increase in SHBG (sex hormone-binding globulin) levels was observed following probiotic therapy and these changes were also statistically significant. Zou et al. noted a significant increase in SHBG in premenopausal women in their research concerning the link between probiotic consumption and sex steroid hormones changes. Additionally, in the same group of women, they also observed a noteworthy estradiol rise [25]. Calcaterra et al., in their narrative review on probiotic usage in adolescents with PCOS and obesity, noted a marked increase in SHBG without any effect on sex steroids [28]. Similar findings were reported by Shamasbi SG et al. in their meta-analysis of reproductive-aged patients with PCOS [30]. No significant impact of probiotics intake on SHBG levels and hormones was detected, as reported by Hu X et al. in their 2023 meta-analysis [31]. Zhang et al. observed a significant decrease in the levels of luteinizing hormone (LH) and the LH/FSH ratio, along with a marked increase in the levels of sex hormones and intestinal short-chain fatty acids (SCFAs) in PCOS patients after supplementation with the probiotic Bifidobacterium lactis V9. The authors suggest that Bifidobacterium lactis V9 promotes the growth of SCFA-producing microbes, which may influence the secretion of gut–brain mediators and subsequently affect sex hormone secretion via the gut–brain axis. They propose this as a potential mechanism by which the consumption of the probiotic regulates sex hormone levels [32]. This may also be a potential mechanism of probiotics action in our study. Studies investigating the effects of probiotic administration on FSH and estradiol levels mainly focus on women during the menopausal transition. One study demonstrated a significant increase in FSH levels, with no effect on estradiol or cortisol levels following probiotic administration [33]. Other research indicates favorable effects on estrogen concentrations during the menopausal transition [34]. Research shows that the estrobolome, a part of the gut microbiome, plays a role in estrogen metabolism by maintaining bacterial secretion of β-glucuronidase. This enzyme deconjugates estrogens and enables them to be reabsorbed in intestines and transported by circulation to distal sites, limiting adverse effects of hypoestrogenism, especially in hypoestrogenic periods like post-menopause [13,25]. In the current study, we did not observe any significant changes in FSH or estradiol levels following probiotic administration. However, our study focused on a different age group.

Evidence of an increase in testosterone levels due to gut microbiome transfer, combined with direct evidence of testosterone synthesis by bacteria inhabiting the gut, suggests the existence of the “testrobolome” [13]. But the research results are contradictory, and some investigators recorded a decrease or no influence on testosterone levels [25,28,30]. We observed a significant decrease in testosterone levels after probiotic administration.

Nevertheless, the effect of probiotics on the hypothalamic–pituitary–adrenal axis and the subsequent reduction in cortisol levels is widely documented [14,15,35]. This has led to the consideration of probiotics as a potential anti-stress therapy both in animal models [36,37] and in humans [18,38]. However, most research has focused on general stress management rather than specific conditions like congenital adrenal hyperplasia (CAH), which involves a genetic condition affecting adrenal hormone production. We haven’t found any information regarding the effects of probiotics on women with CAH. Our study demonstrated a reduction in 17-hydroxyprogesterone levels following probiotic intake on the borderline of statistical significance. However, baseline hormone concentrations were low, ruling out congenital adrenal hyperplasia in all patients. The study does not provide an answer as to whether the same results would apply to patients with CAH. Thus, probiotics’ role in conditions like CAH remains unclear, and research is needed to explore this area.

The direct impact of probiotics on thyroid function remains inadequately established as well. However, there is a theoretical connection between the gut and thyroid through the “gut-thyroid axis.” A healthy gut microbiome may influence the metabolism of thyroid hormones, as certain gut bacteria are involved in the conversion of thyroxine (T4) into the active form, triiodothyronine (T3). Additionally, a well-functioning gut barrier is crucial for optimal immune system activity and the absorption of essential nutrients for thyroid health, such as iodine, selenium, and zinc. Probiotics may improve the composition of the gut microbiota, reduce intestinal inflammation, and enhance nutrient absorption, potentially offering indirect support for thyroid function [39,40,41]. The recent meta-analysis by Shu et al. revealed no significant changes in TSH, fT4, or fT3 levels following probiotic supplementation. However, a positive effect was observed in patients with Graves’ disease, evidenced by a significant reduction in thyroid-stimulating hormone receptor antibody levels [42]. In patients with primary hypothyroidism, the meta-analysis after probiotic bacterial species indicated a nonsignificant decrease in TSH levels, with no effect on fT3 levels. Additionally, no significant changes were observed in fT4 levels, thyroid autoantibodies, levothyroxine doses, or the severity of symptoms, suggesting that probiotics may offer little to no benefit in these patients [12]. Our study does not confirm these findings. Although patients with thyroid disorders were not included in the study and the studied thyroid stimulating hormone (TSH) was within a normal ranges in all patients, we observed significant changes in TSH levels following 12-week probiotic intake. Further studies in patients with PCOS and thyroid dysfunction would be needed to draw broader conclusions. It is likely that the differences in the achieved effects result from the composition of the probiotic (diversity of strains, number of cells), the time of intervention, and the diet used.

There is limited research on the effect of probiotic intake on prolactin levels. One of the studies revealed that in women with polycystic ovary syndrome and hyperprolactinemia who were receiving metformin, the addition of probiotics to metformin therapy significantly reduced prolactin levels compared to metformin therapy alone [43]. In patients with PCOS with prolactin within normal ranges, Zhang et al. did not see signs of a significant change in prolactin concentration after intake of Bifidobacterium lactis V9 probiotic [32]. In our study we observed similar results after 12 weeks of supplementation with a combination of nine bacterial strains of Bifidobacterium and Lactobacillus. In an animal model, Kim et al. observed downregulation of the serum levels of prolactin as a result of administration Lactobacillus helveticus HY7801 (HY7801) in mice with hyperprolactinemia [44]. The aforementioned conflicting study results highlight the need for further research on this subject.

The data regarding the impact of probiotic therapy on BMI is ambiguous. The meta-analysis, published in 2018, pointed out that probiotics administration resulted in a significant reduction in bodyweight in patients who are overweight or obese [45], similar to other studies [26,28]. Our current study concerned PCOS patients of reproductive age who were overweight (BMI greater than or equal to 25 to 29.9 kg/m^2^, according to the National Institute of Health (NIH) and the World Health Organization (WHO) [27]. We observed a significant decrease in BMI only in the probiotic group, with no such change observed in the placebo group. These findings are in contrast to those of the other researchers, who concluded that probiotics are not effective for weight loss [46]. Similar results to our current findings were reported after probiotic supplementation in peri- and postmenopausal women. In that study however, a significant reduction in BMI was also observed in the placebo group, likely due to the healthy, norm caloric diet followed by the participants throughout the study [33].

In summary, our study confirms the significant role of probiotics in hormonal regulation in women with PCOS. We noticed a particularly significant reduction in LH ad androstenedione concentrations, just like the growth of SHBG, which may have a positive impact on these women’s health. Supplementation with the probiotic used in our study may be particularly beneficial for women with PCOS who have reduced SHBG protein synthesis. Therefore, women with a high free androgen (FAI) phenotype may, in our opinion, benefit the most. Following other scientific publications on patients with PCOS, we observed the positive effects of probiotic therapy in reducing BMI in these women.

### Strengths and Limitations

The strength of this study includes its robust design, characterized by a randomized, placebo-controlled, double-blind intervention. However, a major limitation is the relatively small sample size, as participants were selected based on rigorous inclusion and exclusion criteria. Additionally, we did not account for dietary measures or control for the consumption of probiotic-rich fermented foods, such as yogurt, buttermilk, kefir, and fermented products such as tofu, kimchi, beets, cucumbers, and cabbage. To validate our findings, future studies should involve longer intervention durations and larger sample sizes. Given these limitations, the results should be interpreted with caution. As the experiment was terminated after 12 weeks of probiotic therapy, it is not possible to determine whether this improvement was permanent or temporary.

## 5. Conclusions

We have demonstrated the efficacy of 12 weeks of probiotic supplementation containing nine strains (Lactobacillus and Biffidobacterium) on increasing SHBG levels and decreasing LH and androstenedione levels in patients with PCOS during the reproductive period, although such conclusions should be made with caution. Additionally, we observed a significant post-probiotic TSH drop. Probiotic supplementation may be an alternative supporting treatment, especially in the PCOS phenotype of women with a high FAI index.

## Figures and Tables

**Table 1:** Baseline characteristic of the groups.

Parameters	Probiotics Group (n = 19) Mean ± SD	Placebo Group (n = 24) Mean ± SD	*p* Value *
Age [years]	28.47 ± 5.87	28.37 ± 5.38	0.95
Weight [kg]	76.19 ± 17.83	80.54 ± 15.13	0.45
Height [m]	1.66 ± 0.05	1.68 ± 0.05	0.31
BMI [kg/m^2^]	28.47 ± 5.59	28.67 ± 5.45	0.91

BMI—Body Mass Index; SD—standard deviation; * independent *t*-test.

**Table 2:** The within-group comparison of means in both the study and the placebo group before and 12 weeks after intervention.

Variable	Probiotics Group (n = 19)		*p* Value	Placebo Group (n= 24)		*p* Value
	Baseline	After 12 Weeks		Baseline	After 12 Weeks	
FSH (mIU/mL)	5.210	5.400	0.8721	6.115	5.600	0.9539
LH (mIU/mL)	**9.110**	**5.320**	**0.0218**	8.955	8.400	0.3458
Estradiol(pg/mL)	46.500	44.100	0.4688	40.450	44.800	0.3914
Prolactin(ng/mL)	10.300	10.100	0.0836	9.875	9.850	0.6682
TSH (μIU/mL)	**1.820**	**1.320**	**0.0442**	1.305	1.425	0.2904
Androstendione(ng/mL)	**3.525**	**3.200**	**0.0038**	2.535	2.675	0.8303
Testosterone (ng/mL)	0.435	0.440	0.0705	0.379	0.424	0.3606
SHBG (nmol/L)	**37.800**	**48.900**	**0.0012**	43.050	44.500	0.3606
DHEA-S (μg/dL)	294.000	299.000	0.8464	317.500	319.000	0.0574
17(OH)P (ng/mL)	1.230	0.970	0.0502	1.025	0.990	0.0946
BMI (kg/m^2^)	**28.38**	**27.36**	**0.0198**	27.85	27.02	0.2959

Wilcoxon test; FSH—follicle stimulating hormone; LH—luteinizing hormone; TSH—thyroid stimulating hormone; SHBG—sex hormone binding globulin; DHEA-S—dehydroepiandrosterone sulfate; 17(OH)P—17-hydroxyprogesterone; BMI—Body Mass Index. Bold numbers- mean statistically important

**Table 3:** Comparison of the pre-to-post delta changes between the study group and the placebo group.

Variable	Probiotics Group (n = 19)	Placebo Group (n = 24)	*p* Value
	Delta Median Change from Baseline	Delta Median Change from Baseline	
FSH (mIU/mL)	−0.120	−0.465	0.4338
LH (mIU/mL)	**−2.750**	**−0.650**	0.0915 *one-tailed test:* **0.0458**
Estradiol (pg/mL)	−2.100	4.450	0.8545
Prolactin (ng/mL)	−0.890	−0.500	0.4196
TSH (μIU/mL)	**−0.350**	**0.200**	**0.0269**
Androstendione (ng/mL)	**−0.345**	**−0.095**	**0.0262**
Testosterone (ng/mL)	−0.062	0.011	0.1039
SHBG (nmol/l)	**6.200**	**3.850**	**0.0114**
DHEA-S (μg/dl)	5.000	9.500	0.5655
17(OH)P (ng/mL)	−0.110	−0.070	0.4339
BMI (kg/m^2^)	−0.387	−0.086	0.7044

Mann–Whitney U test; FSH—follicle stimulating hormone; LH—luteinizing hormone; TSH—thyroid stimulating hormone; SHBG—sex hormone binding globulin; DHEA-S—dehydroepiandrosterone sulfate; 17(OH)P—17-hydroxyprogesterone; BMI—Body Mass Index. Bold numbers- mean statistically important

## Data Availability

The original contributions presented in this study are included in the article. Further inquiries can be directed to the corresponding author.
